# Improved Regional Homogeneity in Chronic Insomnia Disorder After Amygdala-Based Real-Time fMRI Neurofeedback Training

**DOI:** 10.3389/fpsyt.2022.863056

**Published:** 2022-06-30

**Authors:** Zhonglin Li, Jiao Liu, Bairu Chen, Xiaoling Wu, Zhi Zou, Hui Gao, Caiyun Wang, Jing Zhou, Fei Qi, Miao Zhang, Junya He, Xin Qi, Fengshan Yan, Shewei Dou, Li Tong, Hongju Zhang, Xingmin Han, Yongli Li

**Affiliations:** ^1^Department of Radiology, Henan Provincial People's Hospital and People's Hospital of Zhengzhou University, Zhengzhou, China; ^2^Department of Nuclear Medicine, First Affiliated Hospital of Zhengzhou University, Zhengzhou, China; ^3^Henan Medical Key Laboratory of Molecular Imaging, Zhengzhou, China; ^4^Department of Medical Imaging, Fifth Affiliated Hospital of Zhengzhou University, Zhengzhou, China; ^5^Department of Nuclear Medicine, Henan Provincial People's Hospital and People's Hospital of Zhengzhou University, Zhengzhou, China; ^6^Henan Key Laboratory of Imaging and Intelligent Processing, PLA Strategic Support Force Information Engineering University, Zhengzhou, China; ^7^Health Management Center, Henan Provincial People's Hospital and People's Hospital of Zhengzhou University, Zhengzhou, China; ^8^Department of Neurology, Henan Provincial People's Hospital and People's Hospital of Zhengzhou University, Zhengzhou, China

**Keywords:** chronic insomnia disorder, regional homogeneity, real-time, fMRI, neurofeedback, amygdala

## Abstract

**Background:**

Chronic insomnia disorder (CID) is a highly prevalent sleep disorder, which influences people's daily life and is even life threatening. However, whether the resting-state regional homogeneity (ReHo) of disrupted brain regions in CID can be reshaped to normal after treatment remains unclear.

**Methods:**

A novel intervention real-time functional magnetic resonance imaging neurofeedback (rtfMRI-NF) was used to train 28 CID patients to regulate the activity of the left amygdala for three sessions in 6 weeks. The ReHo methodology was adopted to explore its role on resting-state fMRI data, which were collected before and after training. Moreover, the relationships between changes of clinical variables and ReHo value of altered regions were determined.

**Results:**

Results showed that the bilateral dorsal medial pre-frontal cortex, supplementary motor area (SMA), and left dorsal lateral pre-frontal cortex had decreased ReHo values, whereas the bilateral cerebellum anterior lobe (CAL) had increased ReHo values after training. Some clinical scores markedly decreased, including Pittsburgh Sleep Quality Index, Insomnia Severity Index, Beck Depression Inventory, and Hamilton Anxiety Scale (HAMA). Additionally, the ReHo values of the left CAL were positively correlated with the change in the Hamilton depression scale score, and a remarkable positive correlation was found between the ReHo values of the right SMA and the HAMA score.

**Conclusion:**

Our study provided an objective evidence that amygdala-based rtfMRI-NF training could reshape abnormal ReHo and improve sleep in patients with CID. The improved ReHo in CID provides insights into the neurobiological mechanism for the effectiveness of this intervention. However, larger double-blinded sham-controlled trials are needed to confirm our results from this initial study.

## Introduction

Chronic insomnia disorder (CID) is a highly prevalent sleep disorder, especially with the spread of coronavirus disease 2019 around the world in recent years (1). According to reports, the prevalence of insomnia among American adults is 18.8% and that among the general population in China is 15% (2). The typical symptom of CID is difficulty in falling asleep at bedtime, frequent awakening in the middle of the night, and waking up too early in the morning (1). Long-term insomnia or low sleep quality causes imbalance in the interaction between sympathetic and parasympathetic nerves, resulting in anxiety, depression, and other negative emotions (1, 3). The neurobiological mechanism of CID remains unclear, thus affecting the development of therapeutic methods (4). Therefore, the neurobiological mechanisms should be urgently identified, and innovative therapies of CID should be studied.

Recently, resting-state functional magnetic resonance imaging (rs-fMRI) is an increasingly recognized technique to investigate functional alterations in patients with CID, and this method has unique advantages in clinical research (3, 4). Different methods have been applied to determine the disruptions of the brain activity in CID by using rs-fMRI, including seed-based functional connectivity (FC) (5), independent component analyses (ICA) (6), voxel-mirrored homotopic connectivity (VMHC) (7), amplitude of low-frequency fluctuations (ALFFs) (8), and regional homogeneity (ReHo) (2, 9, 10). However, FC is a hypothesis-driven method, and the definition and exact placement of ROI seeds can be somewhat arbitrary, thus introducing potential biases in the assessment results and impeding the discovery of unexpected regions of interest (11). For ICA, the determination of the generated components with an optimal number is relatively arbitrary, thereby greatly influencing the number of connectivity patterns that can be obtained and producing considerable variations among studies (11). In addition, VMHC focuses on exploring the differences in homotopic coordination rather than the whole brain (12). ReHo and ALFF are both data-driven methods that reflect spontaneous neuronal activity from different perspectives. However, some studies have recognized that ReHo analysis achieves better performance in depict clinical trait than ALFF (13). Therefore, ReHo analysis could be used to gain insight into the neural mechanisms underlying CID.

Resting-state regional homogeneity measures the similarity or synchronicity of the time series of nearest neighboring voxels and reflects the strength of local spontaneous neural activity in the brain (14). It has been successfully applied to reveal abnormalities in the brain function of patients with CID (2, 9, 10). Earlier, Dai et al. (9) found increased ReHo in the left fusiform gyrus and lower ReHo in the bilateral cingulate gyrus and right cerebellum anterior lobe (CAL) in patients with CID. The correlations between the clinical measurements and ReHo value of the fusiform gyrus and frontal lobe were observed (9). Wang et al. (10) discovered abnormal ReHo activities in multiple brain regions, especially in emotion-related areas in patients with CID. Significant correlations were observed between ReHo values of altered brain regions and clinical scores (10). Zhang et al. (2) found that ReHo alterations in the left inferior occipital gyrus may play an important role in the dysfunctional beliefs and attitudes about sleep in patients with CID. Therefore, ReHo is a highly effective and sensitive method for mapping disrupted neural activity to reflect the underlying neurobiological mechanism of CID. However, whether the ReHo of disrupted brain regions in patients with CID can be reshaped to normal after treatment remains unclear.

A novel intervention real-time fMRI neurofeedback (rtfMRI-NF), which has no known side effects and potential longer-term neuroplastic effects, can train people to autonomously regulate brain activity and has huge application prospect in improving cognition or curing diseases (15–20). In comparison with other neurofeedback techniques, such as EEG and non-invasive physical stimulation techniques (transcranial magnetic stimulation), rtfMRI-NF has the advantage of higher spatial resolution and better access to deep relevant brain structures (15–17). Subjects can use rtfMRI-NF to regulate the activity of local brain regions or the functional connections of multiple brain regions to improve clinical symptoms, including depression, anxiety, schizophrenia, and other diseases (18–20). Spiegelhalder et al. (3) found that rtfMRI-NF may be useful for future studies on the treatment of patients with CID. Therefore, we aimed to verify whether the abnormal local ReHo of patients with CID can be reshaped to normal by rtfMRI-NF training.

A target brain region, which is closely related to the pathogenesis of CID, was needed for rtfMRI-NF training (15–18). Multiple local and overall dysfunctions occur in patients with CID, and these dysfunctions are mainly concentrated in the amygdala and emotion and cognition-related brain areas (3, 5, 21, 22). CID is associated with increased amygdala responsiveness to negative stimuli, and its treatment may benefit from strategies that modulate its association with emotion (22). Huang et al. discovered decreased FC between the amygdala and insula, striatum, and thalamus. They also found increased FC of the amygdala with the pre-motor and sensorimotor cortex (5). The amygdala, which is positioned in the limbic system's center and plays a critical role in the formation, expression, and perception of unpleasant emotions, has been implicated in the important pathophysiology of CID (23). Previous studies supported a functional dissociation between the left and right amygdala in terms of temporal dynamics. The right amygdala is engaged in the rapid and automatic detection of emotional stimuli, whereas the left amygdala participates in more detailed and elaborate stimulus evaluation (24, 25). Moreover, many researchers have successfully used rtfMRI-NF training to help patients regulate the activity of their left amygdala through positive autobiographical memory to change brain function and clinical symptoms (16–18). As a result, targeting the activity of the left amygdala for rtfMRI-NF regulation may improve the sleep of patients with CID, thereby providing a new breakthrough point for investigating the neurological mechanism.

Taken together, amygdala-based rtfMRI-NF training could reshape resting-state local spontaneous neural activity accompanying improvement of sleep in patients with CID. To prove our hypothesis, we used rtfMRI-NF to train patients with CID to regulate the activity of the left amygdala for three sessions in 6 weeks. The rs-fMRI data were collected before and after training. Then, the ReHo method was used to explore the effect of rtfMRI-NF training. We also investigated the relationships between the ReHo value of altered regions and the changes of clinical variables.

## Materials and Methods

### Participants

The study design and patient approval for this study were granted by the Ethics Committee of the Henan Provincial People's Hospital. All patients with CID were recruited by advertisement or introduction of hospital doctors from January 2018 to December 2021. All patients signed an informed consent form and were compensated for their participation. All participants underwent a comprehensive neuropsychological and clinical assessment, including Pittsburgh Sleep Quality Index (PSQI) (26), Insomnia Severity Index (ISI) (27), Hamilton Depression Scale (HAMD) of 17 items (28), Beck Depression Inventory (BDI) (29), and Hamilton Anxiety Scale (HAMA) of 14 items (30). To exclude the potential influence of drugs, the patients were asked not to take medications or other treatment methods for insomnia from 2 weeks before the experiment to the end of training. Patients who cannot bear the torture of insomnia can quit at any time. Based on the Fifth Edition of Diagnostic and Statistical Manual of Mental Disorders diagnostic criteria (DSM-V), the inclusion criteria are as follows: (1) age of 18–70 years; (2) right-hand dominance and native Chinese speaker; (3) fatigue, irritability, cognitive decline, and other insomnia symptoms should last for at least 3 months; (4) PSQI score ≥8; (5) no history of psychiatric or neurological disorders (e.g., schizophrenia, stroke); (6) no any secondary sleep problems (e.g., restless leg syndrome, obstructive sleep apnea); (7) no history of alcohol and substance abuse or dependence; and (8) no brain lesions or prior substantial head trauma found by T2-weighted dark-fluid and T1-weighted MR images. Considering the concomitant relationship between CID and depression and anxiety (31, 32), this study did not limit the depression and anxiety score of patients with CID when recruiting them. In addition, an overnight polysomnography (PSG) was used to exclude participants with occult sleep disorders. The PSG was recorded using an ambulatory recording system (Compumedics Siesta, Australia). The collected PSG parameters included total sleep time (TST), sleep efficiency (SE), sleep onset latency (SOL), and number of awakenings (NOA).

### Power Calculation

The sample size was calculated using the G^*^Power software (33). Considering that no relevant research has been published, the parameters of effect size were set at 0.5 to estimate the sample size (18). To achieve 80% power at *P* = 0.05 in the two-sided (*t*-test) test, a sample size of *n* = 34 participants was required. Considering a maximum possible dropout rate of 20%, 41 subjects were needed. Given that this clinical trial of fMRI-NF was conducted as a pilot study to test the feasibility, the performed power calculation was of limited value because it is based on effect sizes (18). Furthermore, fMRI-NF is still in the early phases of clinical evaluation, and it is a relatively complex and time-consuming process (18). Finally, 33 patients with CID completed all experimental procedures. However, four participants were excluded because of excessive head movements during resting-state scanning. One subject dropped out because of the pause during training.

### Procedure

**Figure 1:**
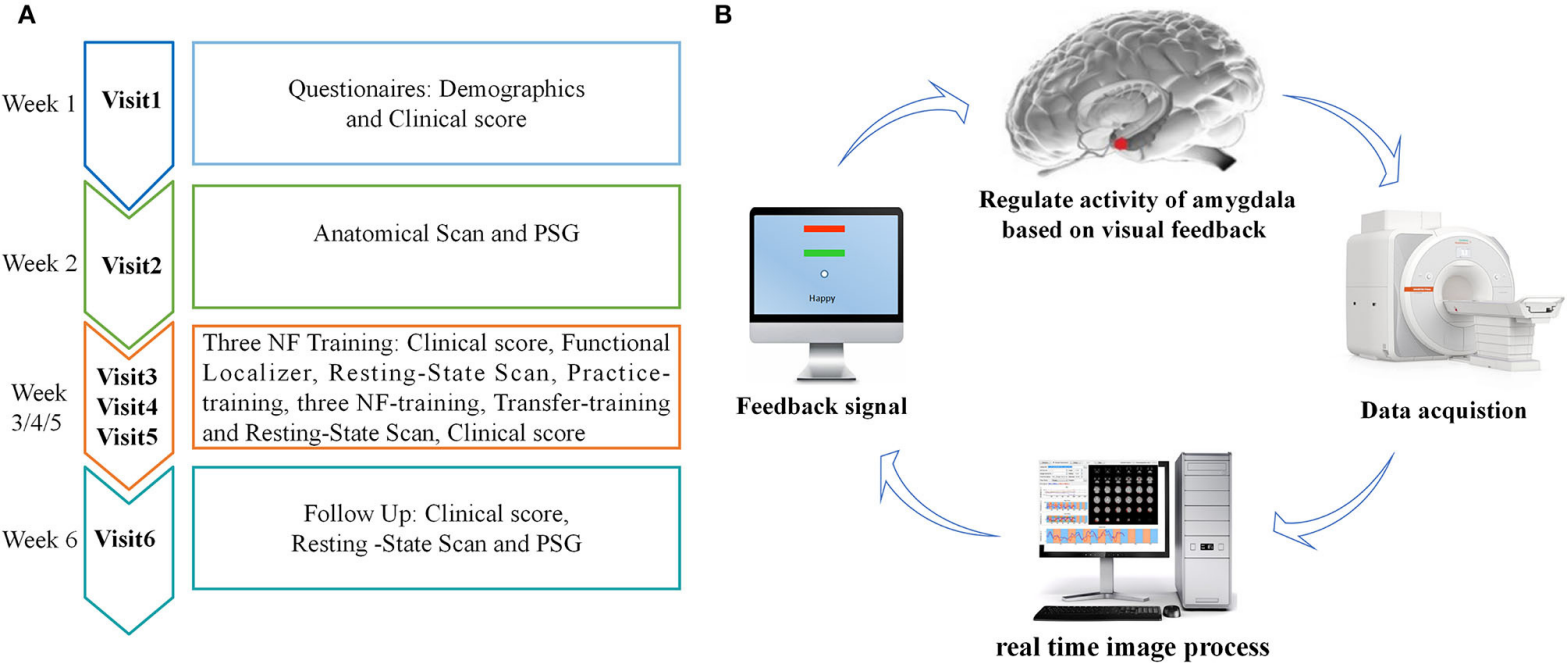
**(A)** Procedure of rtfMRI-NF training experiment. The experimental protocol consisted of six visits, lasting for ~6 weeks. During the third, fourth, and fifth visits, each rtfMRI-NF training session included eight runs, lasting for ~50 min. **(B)** Schematic of amygdala-based rtfMRI-NF loop for patients with CID. Typically, brain images were acquired using the MR scanner online and analyzed using the OpenNFT software in real time. Then, the signal of amygdala activity was provided as feedback to patients with CID for neural self-regulation. rtfMRI-NF, real-time fMRI neurofeedback; PSG, polysomnography; CID, chronic insomnia disorder.

The experimental procedure of rtfMRI-NF training is shown in Figure 1A. The participants completed the experiment by visiting six times with 1 week internal. During the first visit, we gathered information on broad demographic parameters. The PSQI, ISI, HAMD, BDI, and HAMA were also provided by the participants. During the second visit, the participants completed MRI scans and familiarized themselves with the MRI scanning environment. To rule out any structural abnormalities in the brain, we collected routine axial T2-weighted dark-fluid and T1-weighted MR images. To register the template of the amygdala from the standard space to the subject space for real-time processing data during training, we collected high-resolution T1-weighted structural images. Afterward, PSG was performed overnight. During the third, fourth, and fifth visits, the participants completed the same clinical and self-report measures as the first visit and their three rtfMRI-NF training sessions. The detailed paradigm of rtfMRI-NF training is shown in the following section. During the sixth visit, the participants finished overnight PSG as in their second visit. Meanwhile, the participants finished rs-fMRI scans, and the same clinical score as in their third visit was obtained.

### rtfMRI-NF Training Paradigm

The schematic of amygdala-based rtfMRI-NF loop is shown in Figure 1B. The participants were required to write down three or more specific autobiographical memories about themselves. Before rtfMRI-NF training, we informed the patients about the details of experimental process and the specific tasks under different stimuli. The signals of brain activity from the left amygdala region were provided as feedback to them for regulation during training. The amygdala is defined from the Talairach space with a radius of 7 mm and the coordinates (i.e., −21, −5, and −16), and its signal is displayed as temperature bars updating once per reaction time (TR) (2 s) (20, 34). The OpenNFT system was used to perform real-time online data analysis (35). Detailed information about the steps and parameter are provided in the article published by Koush et al. (35). Eight runs were carried out in each training session lasting for ~50 min, including the functional localizer, resting-state scan, practice-training, three NF-training, transfer-training, and resting-state scan. The functional localizer run was carried out for 20 s, and the resting-state scan run was carried out for 7 min. The five remaining runs were carried out for 6 min and 50 s. In the two resting-state scan runs, we collected rs-fMRI data of the participants, who were instructed to fix on green cross, remain awake, and think of nothing in particular. The practice-training and transfer-training runs shared the same paradigms with NF training but without a feedback signal. The participants were allowed to familiarize with the rtfMRI-NF training procedure by designing the practice-training run. To test whether participants had mastered the regulation strategy, we added a transfer training run after three NF training runs. Each NF training run consisted of alternating 30 s rest and 30 s happy blocks, with seven blocks of rest and six blocks of happy. At rest blocks, participants were asked to stare at the green cross on the screen to calm their mind. During happy blocks, the participants were instructed to increase the height of thermometer on the screen by recalling a positive autobiographical memory.

### Data Acquisition

A MAGNETOM Prisma 3T MR scanner (Siemens Healthcare, Erlangen, Germany) with a 64-channel head-neck coil was used for fMRI data acquisition at the Medical Imaging Center of our hospital. Earplugs and foam pads were used to minimize scanner noise and head motion. A medical tape was fixed on participants' foreheads to help them control their movements. rs-fMRI data were acquired using an echo-planar imaging sequence with 210 volumes lasting for 420 s. The corresponding acquisition parameters were set as follows: TR: 2,000 ms, echo time (TE): 30 ms, field of view (FOV): 224 mm × 224 mm, matrix size: 112 × 112, slices: 27, slice thickness: 4 mm, gap: 1 mm, and flip angle: 90°. High-resolution T1-weighted structural images were acquired with the following parameters: TR: 2,300 ms, TE: 2.27 ms, FOV: 250 mm × 250 mm, matrix size: 256 × 256, slices: 192, slice thickness: 1 mm, and flip angle: 8°.

### Data Processing

The rs-fMRI data collected in Visit 6 and the first scan of Visit 3 (Figure 1A) were used for analysis in this study. Data pre-processing and ReHo analysis were carried out by using the Data Processing and Analysis of Brain Imaging (DPABI, http://rfmri.org/DPABI) toolbox (36). First, we discarded the first 10 volumes of each run for signal stabilization and participant adaptation. Then, slice timing and head-motion correction were carried out. Data with maximum displacement in head rotation of larger than 2° or any directions of larger than 2 mm were excluded from further analysis. For precise spatial normalization of the fMRI data, individual high-resolution T1-anatomic images were registered to the mean fMRI data, and the resulting aligned T1-weighted images were segmented and transformed into standard Montreal Neurological Institute space by using the DARTEL toolbox. Furthermore, white matter, cerebral-spinal fluid signals, and 24-head realignment parameter were regressed out as covariates. Subsequently, regressed functional images were specially normalized to the group template by using the transfer parameter estimated by DARTEL segmentation and resampled to 3 × 3 × 3 mm^3^ voxels. Finally, linear trend and temporal band-pass filtering (0.01–0.1 Hz) was applied to reduce low-frequency drift and physiological high-frequency respiratory and cardiac noise.

### ReHo Calculation

Regional homogeneity maps were generated on the pre-processed rs-fMRI data as previously described (2, 9, 10). The Kendall's coefficient of concordance (KCC) was calculated to measure the similarity of the time series of a given voxel to those of its nearest 26 voxels. To reduce the influence of individual variation in KCC values, we performed ReHo map normalization by dividing the KCC of each voxel by the averaged whole-brain KCC. Finally, the ReHo maps were spatially smoothed using a Gaussian kernel with 6 mm full-width at half-maximum. A group template of 90% was generated for fMRI processing and statistics. To assess the effect of amygdala-based rtfMRI-NF training on the CID brain, we compared the whole-brain ReHo differences between after training and before training conditions *via* the paired *t*-test. The Gaussian random field (GRF) theory correction procedure was used for multiple comparisons.

### Statistical Analysis

#### Demographic and Clinical Data Analysis

The data of the clinical score analyzed were collected during the sixth visit and at the beginning of the third visit. The PSG data analyzed were collected during the sixth and second visits. The distribution of clinical data was tested using the Kolmogorov-Smirnov method. Continuous variables with normal distribution were analyzed using the independent paired *t*-test and expressed as mean ± standard deviation. Otherwise, the Wilcoxon signed-rank test was used to analyze non-normally distributed data, which were expressed as median and interquartile range. All statistical analyses were performed using SPSS version 22.0 (http://www.spss.com; Chicago, IL). The threshold for statistical significance was set at *P* < 0.05, and all hypothesis tests were two-tailed.

#### Brain-Behavior Correlation Analysis

Based on the paired *t*-test, the altered ReHo brain regions were located. Then, the mean ReHo values of these brain regions in after training condition were extracted. After this, partial correlation was performed to examine the association between the values of ReHo and the changes in the clinical scores and indexes of PSG, including PSQI, ISI, HAMD, HAMA, BDI, TST, SE, SOL, and NOA with age, gender, and education as covariates. Statistical significance was considered at *P* < 0.05. Multiple comparison correction was performed by the false discovery rate (FDR).

## Results

### Demographic and Clinical Data

In this article, 28 patients with CID were included for further analysis. The demographic data were as follows: 21 females; education: 13.1 ± 3.3 years; and age: 45.7 ± 13.2 years. The normal distribution of clinical characteristics data included PSQI, ISI, HAMD, HAMA, BDI, and TST. However, the SE, SOL, and NOA non-normally distributed. After rtfMRI-NF training, the PSQI, ISI, BDI, and HAMA of patients with CID showed significant differences (*P* < 0.05, Table 1) compared with those before training. However, HAMD, TST, SE, SOL, and NOL showed no significant differences. Detailed information of these results are shown in Table 1.

**Table 1:** Changes in clinical variables before and after training in patients with chronic insomnia disorder.

**Variables**	**Before training**	**After training**	**T/Z value**	***P*-Value**
PSQI	13.86 ± 3.35	11.25 ± 3.31	4.87	<0.001
ISI	17.82 ± 4.83	14.18 ± 6.14	3.46	0.002
HAMD	19.11 ± 8.01	16.86 ± 7.47	1.76	0.09
BDI	19.29 ± 10.70	15.89 ± 9.25	2.88	0.008
HAMA	19.82 ± 9.45	16 ± 12.15	2.65	0.013
TST (min)	380.11 ± 101.70	404.27 ± 81.22	1.18	0.25
SE (%)	78.75 (66.25–84.78)	78.6 (71.33–89.98)	−0.62	0.539
SOL (min)	20.5 (7.25–58.13)	12 (6.63–27.13)	−1.95	0.052
NOA	20.5 (10.25–26.75)	15 (8–26.5)	−1.49	0.137

*Normally distributed data are expressed as mean ± SD, and P-values were obtained using two-tailed paired t-test. Non-normally distributed data were presented as median and interquartile range, and P-values were obtained using the Wilcoxon signed-rank test. PSQI, Pittsburgh sleep quality index; ISI, insomnia severity index; HAMD, Hamilton depression rating scale; BDI, Beck depression inventory; HAMA, Hamilton anxiety rating scale; TST, total sleep time; SE, sleep efficiency; SOL, sleep onset latency; NOA, number of awakenings*.

### ReHo Analysis

**Figure 2:**
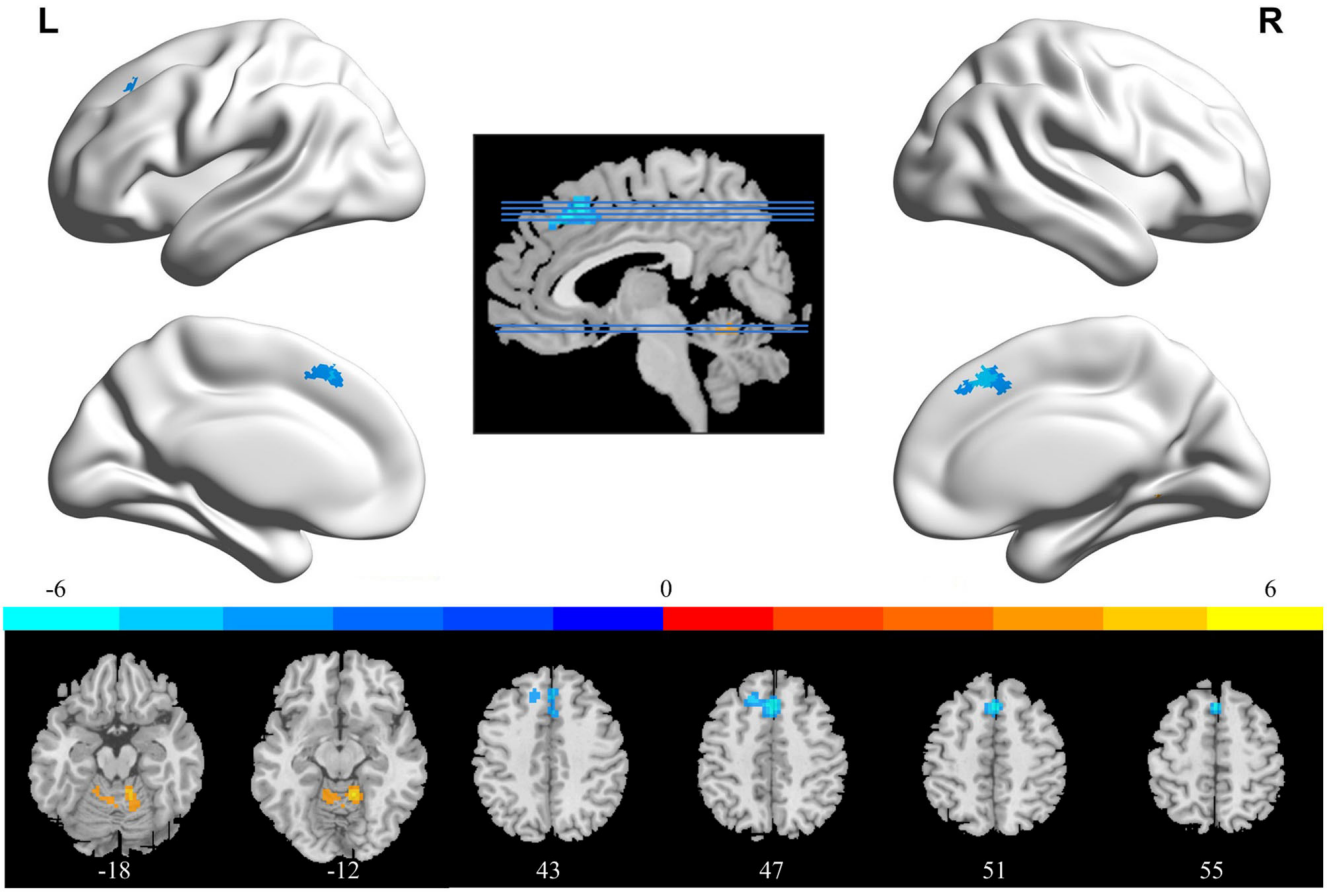
Brain areas that exhibited altered ReHo induced by real-time fMRI neurofeedback training (after training vs. before training). Results were set at voxel-level *P* < 0.001, cluster-level *P* < 0.05, and *t* = 3.29 (Gaussian random field corrected). Warm colors indicate regions in which ReHo increased significantly, whereas cool colors indicate regions in which ReHo decreased significantly. The color bar indicates the *t*-value. The numbers at the bottom indicate the axial slices. ReHo, regional homogeneity; L, left; R, right.

**Figure 3:**
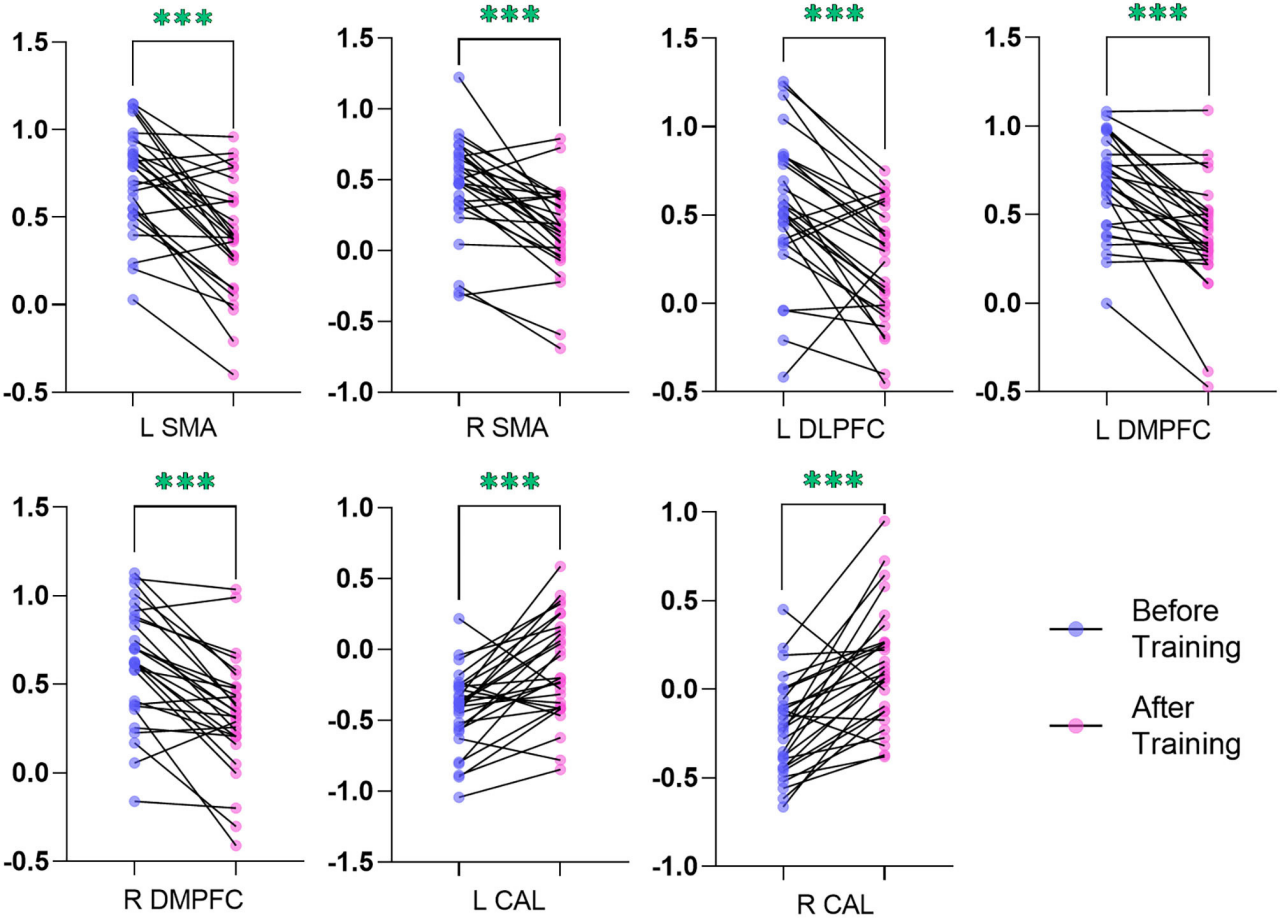
Plot of the z-transform ReHo values in brain regions that exhibited altered ReHo. The blue dots (before training condition) and the pink dots (after training condition) indicated the mean values of ReHo in the different brain regions of all the subjects. L, left; R, right; ReHo, regional homogeneity; SMA, supplementary motor area; DLPFC, dorsal lateral pre-frontal cortex; DMPFC, dorsal medial pre-frontal cortex; CAL, cerebellum anterior lobe. ***indicates *p* < .001.

In comparison with the ReHo before training condition, patients with CID showed decreased ReHo in bilateral dorsal medial pre-frontal cortex (DMPFC), supplementary motor area (SMA), and left dorsal lateral pre-frontal cortex (DLPFC) and increased ReHo in bilateral CAL. Table 2 shows the detailed information on activation centers. The spatial distributions of altered ReHo regions are shown in Figure 2. The results were set at voxel-level: *P* < 0.001, cluster-level: *P* < 0.05, *t* = 3.29, GRF corrected. The distributions of z-transform ReHo values in brain regions that exhibited altered ReHo are shown in Figure 3.

**Table 2:** Brain regions exhibited altered ReHo in patients with chronic insomnia disorder (after training vs. before training).

**Brain regions**	**Whole cluster size**	**Cluster size**	**MNI co-ordinates**			***t* score**
			**x**	**y**	**z**	
Left supplementary motor area	152	43	0	21	48	−6.2269
Left dorsal lateral pre-frontal cortex		35	−15	30	45	−5.3858
Right supplementary motor area		26	3	24	48	−6.3088
Left dorsal medial pre-frontal cortex		23	0	27	48	−5.7855
Right dorsal medial pre-frontal cortex		19	3	30	45	−6.2449
Left cerebellum anterior lobe	137	62	12	−48	−15	6.8476
Right cerebellum anterior lobe		42	−12	−48	−12	4.55

*Results were set at voxel-level: P < 0.001, cluster-level: P < 0.05, t = 3.29 (Gaussian random field corrected). ReHo, regional homogeneity; MNI, Montreal Neurological Institute*.

### Brain Behavior Correlation Analysis

**Figure 4:**
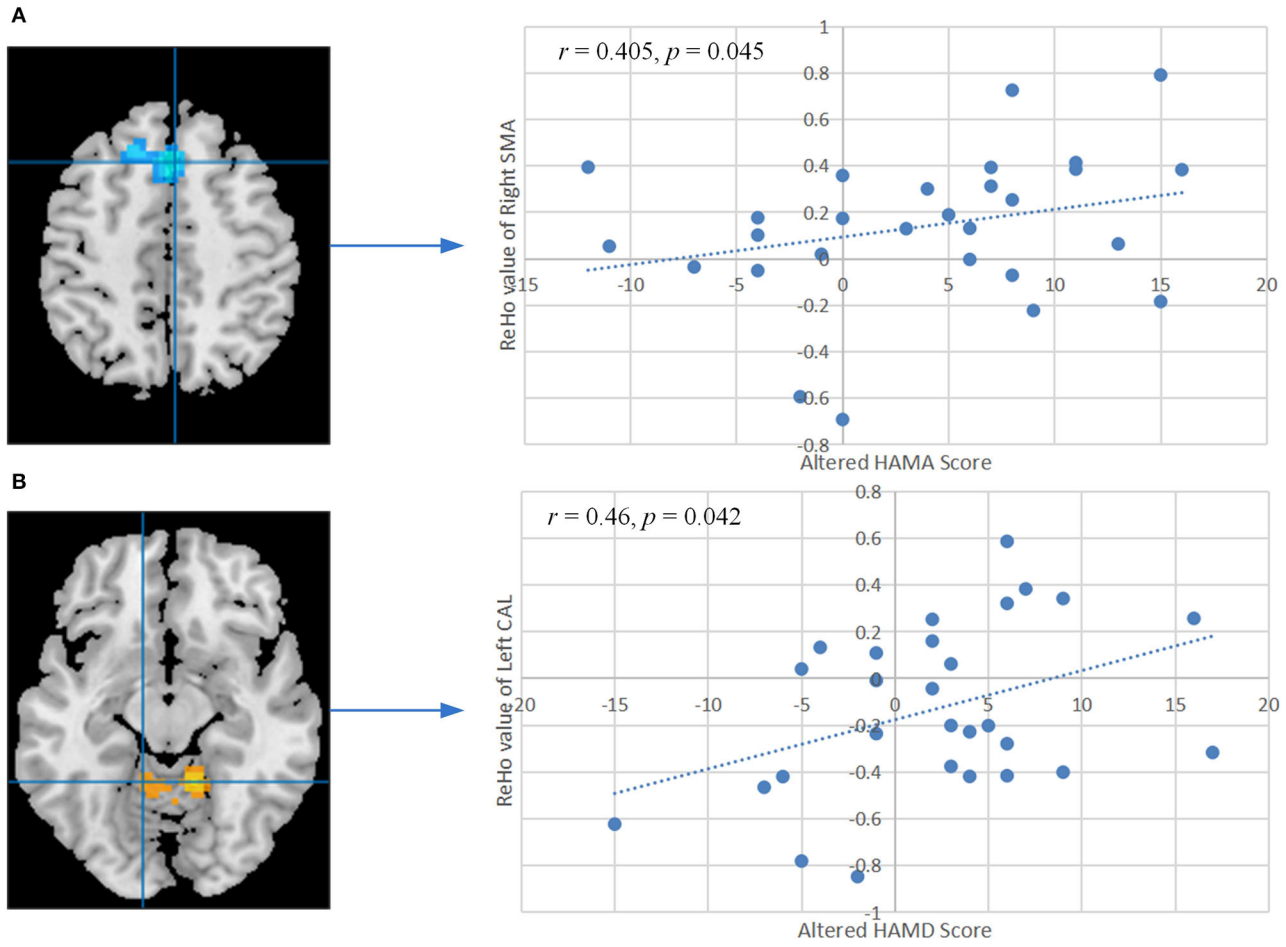
**(A)** Correlation between the altered HAMA score and ReHo values of the right SMA after training. **(B)** Correlation between the altered HAMD score and ReHo values of the left CAL after training. The *P-*values were corrected by the false discovery rate. ReHo, regional homogeneity; HAMA, Hamilton Anxiety Scale; HAMD, Hamilton Depression Scale; SMA, supplementary motor area; CAL, cerebellum anterior lobe.

The ReHo values of right SMA also showed significant positive correlation with the changed HAMA score (*r* = 0.405, *P* = 0.045, FDR corrected, Figure 4A). A remarkable positive correlation was also found in the changed HAMD score (after training minus before training) with ReHo values of the left CAL (*r* = 0.46, *P* = 0.042, FDR corrected, Figure 4B). The changed ISI score and ReHo values of the left CAL showed a positive correlation but did not reach a significant level (*r* = 0.356, *P* = 0.08, Supplementary Figure S1). The ReHo values of the others altered brain regions did not show significant correlation with the other changed clinical scores and indexes of PSG.

## Discussion

In this study, based on the ReHo method, we explored whether the resting-state local spontaneous neural activity in patients with CID could be regulated by amygdala-based rtfMRI-NF training accompanying improvement of clinical symptoms. Results showed that the bilateral DMPFC, SMA, and left DLPFC had decreased ReHo values, and the bilateral CAL had increased ReHo values. Some clinical scores, including PSQI, ISI, BDI, and HAMA, were markedly decreased. Additionally, the ReHo values of the left CAL were positively correlated with the changed HAMD score. The ReHo values of the right SMA also showed remarkably positive correlation with the HAMA score. These results support our hypothesis that amygdala-based rtfMRI-NF training could reshape abnormal ReHo and improve sleep of patients with CID.

The DMPFC, as a key node of default mode network (DMN), is engaged in self-referential mental activity and emotional processing and has more connection with limbic areas such as amygdala (37–39). Koenigs et al. (40) discovered that the sleep initiation and maintenance is related to damage of the left DMPFC. Notably, the intrinsic activity in the DMPFC of the normal sleep subject is reduced during a natural sleep and shifts to a deep sleep (37). Furthermore, Yu et al. (38) reported that the patients with CID showed significantly increased global FC density in the left DMPFC. Besides, core areas of the DMN showed greater activation in patients with CID compared with healthy controls (HCs) in self-referential-related tasks (41). These results support the hypothesis of hyperarousal to explain the etiology and maintenance of CID (3, 4, 38). An increasing number of studies supports that CID may be conceptualized as a disorder associated with the overactivity of certain brain areas of the DMN (42, 43). The widespread hyperarousal of several systems (e.g., cognitive, physiological, and emotional) occurs during insomnia, which consequently prevents relaxation (42). In this study, the ReHo values of the bilateral DMPFC decreased after training. Our findings may reflect that the amygdala-based rtfMRI-NF training could reshape the disrupted ReHo of DMPFC.

Chronic insomnia disorder has a close relationship with depression and anxiety (44, 45). Therefore, our study aimed to improve the sleep of patients with CID by training them to regulate amygdala activity through recalling happy emotions and then improve emotions states. The DMPFC and amygdala are the key structures of cognitive-affective brain systems (46). Insomnia may disrupt reward-related brain function, a potentially important factor in the development of depressive disorder (47). Melynda et al. (47) found that DMPFC response to reward anticipation and depressive symptoms mediates the relationship between early adolescent non-restorative sleep and late adolescent depressive symptoms. Moreover, the activity of DMPFC increased in HCs during a positive emotion regulation task (46). Taken together, our results indicate that the cognitive-affective brain systems in patients with CID could be modulated through amygdala-based rtfMRI-NF training.

The DLPFC is a critical brain region of central executive network (CEN) and is involved in neurocognitive functions, including executive functions, decision-making, working memory, social cognition, and top-down emotional regulation (48, 49). The DLPFC of patients with CID exhibits structural and functional impairment, which may be lead to early rising and difficulty in falling asleep (50–53). In comparison with HCs, two studies observed decreased gray matter volume in the bilateral DLPFC of patients with CID, thus providing basis for functional abnormalities (52, 53). Huang et al. (50) discovered reduced FC strength in the right DLPFC in patients with CID. The global FC density increased in the right DLPFC (38). These results indicated an impairment of intrinsic FC network in patients with CID. In addition, patients with CID showed high FC between the DLPFC and salience network, which had a remarkable significant positive correlation with PSQI score (49). In terms of spontaneous neuronal activity, Liu et al. (54) discovered increased ALFF values in the bilateral DLPFC of patients with major depressive disorder (MDD) and high insomnia and patients with MDD and low insomnia compared with HCs. Zhang et al. (2) discovered increased ReHo values in the right DLPFC of patients with CID.

Notably, decreased ReHo values were found in left DLPFC after training in this research. DLPFC not only plays a major role in cognitive control but also has an intimate relationship with emotional processes (49, 55). Patients with CID also showed increased amygdala reactivity to insomnia-related stimuli and increased levels of anxiety and depression (22, 49). Thus, DPLFC controls amygdala activity in response to external sleep-related emotional stimuli. At rest, people are less responsive to the outside stimuli. However, this function was disrupted in patients with CID and the activity of DLPFC is increased to inhibit excessive emotional response. Our training enhanced the top-down executive controlling of amygdala by DLPFC. Subsequently, the activity of DLPFC was reduced at rest, thus promoting sleep. In this study, the improvement in symptoms of both depression and anxiety in patients with CID also support the influence of rtfMRI-NF training in reducing hyperarousal. Recently, several studies selected DLPFC as a brain target of non-invasive brain stimulation, including transcranial magnetic stimulation (TMS) and transcranial direct/alternating current stimulation (tDCS/tACS), for CID treatment (56–58). The results showed that it could be a good supplement to drugs in the treatment of CID (56–58). Therefore, DLPFC may also be an effective target for rtfMRI-NF training in the treatment of CID, and future studies are needed to verify this hypothesis.

The sensory-motor network (SMN), including SMA, pre-central gyrus, and post-central gyrus, plays a role in the continuous sensory process of environmental stimuli and proprioceptive information (59). FC within SMN is increased in patients with CID (5, 59–61). In comparison with HCs, Huang et al. (5) found increased FC mainly between the amygdala and pre-motor cortex, sensorimotor cortex in patients with CID. This result might reflect an adaptive response to the “internal” threat in patients with CID (5). The difficulty in falling asleep is associated with high FC between primary sensory and SMA in patients with CID (60). By using the graph theory analysis method, our previous study found increased nodal centralities of right pre-central gyrus in patients with CID (61). The node betweenness of the right pre-central gyrus was positively correlated with the PSQI score in patients with CID (61). Wang et al. (10) discovered increased ReHo in the bilateral pre-central gyrus belonging to SMN, and this parameter is significantly correlated with SAS scores. Therefore, the SMA of patients with CID may be hyperactivity and hyperconnectivity. After amygdala-based rtfMRI-NF training, we found decreased ReHo in bilateral SMA in patients with CID. To some extent, this result is consistent with previous findings. After the CID group underwent cognitive-behavioral therapy, response to sleep-related stimuli of left SMA and FC between the left putamen and left SMA decreased (62, 63). The ReHo values of the right SMA also showed a remarkably positive correlation with the changed HAMA score, indicating an improved anxiety state to external environment in patients with CID. In HCs, those with shorter sleep duration show greater impairment of psychomotor ability than those with longer sleep duration, that is, mental movement in the brain increases before falling asleep and during sleep, resulting in light sleep or difficulty falling asleep in patients with CID (64). Patients with CID fall into a perpetual cycle of somatic hyperarousal and increased sensitivity to sensory stimulation, causing further cortical arousal and difficulty in sleep initiation and maintenance (60). Therefore, amygdala-based rtfMRI-NF training may exert its treatment effects on CID by reducing hyperarousal of the SMA to sleep-related threatening stimuli.

The cerebellum is closely related to cognitive and emotional processing and sleep (9, 65). Several neuroimaging studies have reported cerebellar changes in CID (2, 9, 10, 65). A structural neuroimaging reported that the severity of insomnia has a negative correlation with the gray matter volume of the cerebellum (65). In comparison with HC, Dai et al. (9) found that patients with CID showed decreased ReHo values in the right CAL. Recently, Zhang et al. (2) also found decreased ReHo values in left CAL in patients with CID compared with HC. Therefore, disturbed nocturnal sleep may damage the function of the cerebellum and may be associated with disturbed negative mood state in CID (2, 10). In terms of the FC network, Huang et al. (50) discovered a significant reduction in FC strength in the right CAL, which is mainly located in the bilateral basal ganglia/thalamus and bilateral superior frontal gyrus. They pointed out that the disrupted connectivity in the right CAL affects patients with CID *via* the CEN and SMN system (50). However, the diminished activity of CAL was normalized after amygdala-based rtfMRI-NF training, as well as DMPFC in the CEN system and SMA in SMN system. The ReHo values of the left CAL showed a remarkably positive correlation with the changed HAMD score and may reflect the improved emotional state of the patients with CID. CID and depression are intimately related, which may suggest an overlapping neurobiology (1). Sleep disturbance is common in patients with depression, and patients with insomnia are at high risk of developing depression (31). Gebara et al. (32) indicated that treating CID in patients with depression has a positive effect on mood. The results of this article also confirmed this conclusion. Therefore, in the future treatment of CID, the patient's depression needs to be considered at the same time. Additionally, the ReHo values in the left CAL is associated with higher changed ISI score and closely reached significance level. Although this result did not reach significance, we think that this result is meaningful for the exploratory study of rtfMRI-NF in the treatment of CID. Thus, we added it to this report. We speculated that some influencing factors were present, including rtfMRI-NF training duration, participants' cooperation, training specifications, and so on, which affected the clinical changes in ISI and CLA. Hence, this result should be interpreted properly with caution. In summary, amygdala-based rtfMRI-NF training may normalize the activity of CAL to enhance the emotion regulation ability and thus improve sleep.

### Limitations and Strengths

This study has several limitations. We did not include a sham feedback group. Thus, the ReHo changes of brain after amygdala-based rtfMRI-NF training may be attributable to placebo rather than the actual therapeutic effects. Patients with CID that participate in the experiment hope to improve sleep, thereby reducing the suffering of insomnia. Their compliance is greatly reduced, and if they find that the effect is not significant, they may realize that they are in the sham feedback group. Therefore, only a real feedback group was included to ensure the training effect of patients with CID. Accordingly, in future studies, we aim to add three sham feedback sessions before three real feedback sessions. Moreover, the sample size of this study was relatively small. The rtfMRI-NF training is time-consuming and relatively complex, and the enrolled participants are required to meet PSG diagnostic criteria, thus limiting the number of enrolled patients with CID. Therefore, controlled studies with larger sample size are needed to verify our results from this initial study.

## Conclusion

In summary, our study provided an objective evidence that amygdala-based rtfMRI-NF training could reshape abnormal ReHo and improve the sleep in patients with CID. The improved regions with abnormal ReHo are mainly located in DMN, CEN, SMA, and CAL. The improved abnormal spontaneous neural activity in CID provided insights into the neurobiological mechanism for the effectiveness of amygdala-based rtfMRI-NF training. However, double-blinded sham-controlled trials with a larger sample size are needed to confirm our results from this initial study.

## Data Availability Statement

The raw data supporting the conclusions of this article will be made available by the authors, without undue reservation.

## Ethics Statement

The studies involving human participants were reviewed and approved by Ethics Committee of the Henan Provincial People's Hospital. The patients/participants provided their written informed consent to participate in this study.

## Author Contributions

ZL and JL conceived the study, analyzed the data, and wrote the manuscript. BC and XW performed the experiments and wrote the manuscript. ZZ, HG, CW, JZ, FY, and SD designed and performed the experiments. FQ, MZ, JH, XQ, and HZ collected the clinical samples and performed the experiments. LT, XH, and YL conceived the study, designed the experiments, supervised the project, and wrote the manuscript. All authors contributed to the article and approved the submitted version.

## Funding

This study was supported by the National Natural Science Foundation of China (82071884), Science and Technology Project of Henan Provincial Science and Technology Department (222102310198), Young and Middle-aged Health Science and Technology Innovative Talent Cultivation Project of Henan Provincial Leading Talents (YXKC2020004), and Henan Province Medical Science and Technology Research Project (LHGJ20200060).

## Conflict of Interest

The authors declare that the research was conducted in the absence of any commercial or financial relationships that could be construed as a potential conflict of interest.

## Publisher's Note

All claims expressed in this article are solely those of the authors and do not necessarily represent those of their affiliated organizations, or those of the publisher, the editors and the reviewers. Any product that may be evaluated in this article, or claim that may be made by its manufacturer, is not guaranteed or endorsed by the publisher.
