# Supplementary material for: Improved Regional Homogeneity in Chronic Insomnia Disorder After Amygdala-Based Real-Time fMRI Neurofeedback Training
Source: Front Psychiatry. 2022 Jun 30;13:863056. doi: 10.3389/fpsyt.2022.863056 (PMC9279663; doi:10.3389/fpsyt.2022.863056)
Supplement: Supplementary file 1 [file Table_1.DOCX]

**Supplementary Materials**

**
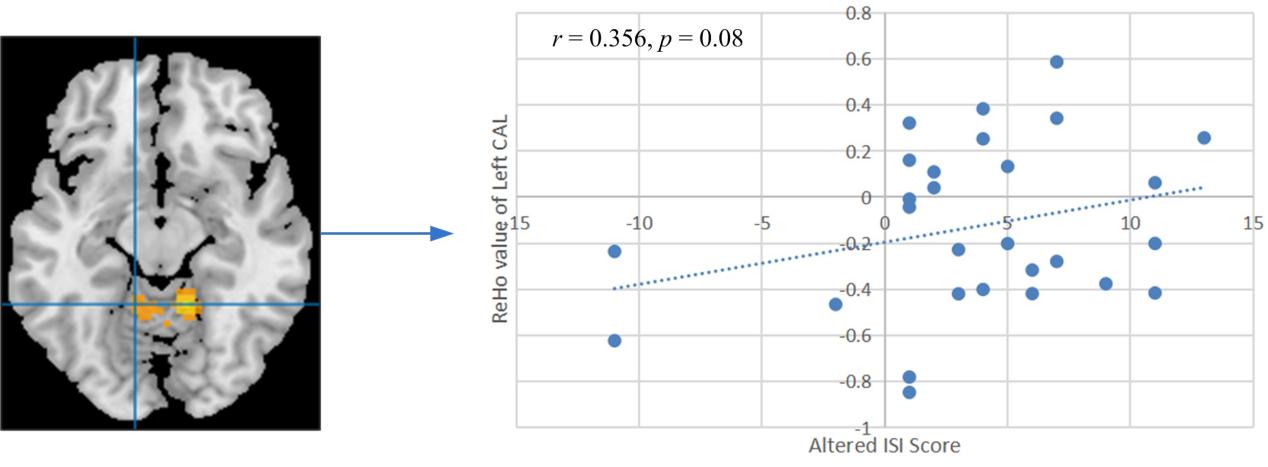
**

**Figure S1.** Correlation between the altered ISI score and regional homogeneity values of the left CAL after training. Abbreviations: ISI, Insomnia Severity Index; CAL, cerebellum anterior lobe.
